# Syphilis Cases Among Pregnant Women and Newborns in the Military Health System, 2012–2022

**Published:** 2024-12-20

**Authors:** Katherine S. Kotas, Shauna L. Stahlman, Saixia Ying, David H. Yun, Charles E. McCannon, John F. Ambrose

**Affiliations:** 1 Disease Epidemiology Program, Defense Centers for Public Health–Aberdeen, Defense Health Agency, Aberdeen Proving Ground, MD; 2 Epidemiology and Analysis Branch, Armed Forces Health Surveillance Division, Silver Spring, MD; 3 Defense Centers for Public Health–Falls Church, Armed Forces Health Surveillance Division, Falls Church, VA

## Abstract

This report presents the rates of maternal syphilis among pregnant women and congenital syphilis among newborns in the Military Health System (MHS) beneficiary population from 2012 to 2022. Medical encounter data from military hospitals and clinics as well as civilian health care facilities were obtained from the Defense Medical Surveillance System (DMSS) to determine pregnancies, live births, and confirmed diagnoses of maternal and congenital syphilis. The rate of maternal syphilis in female MHS beneficiaries increased by 233% between 2012 (n=123, 66.0 per 100,000 births) and 2022 (n=169, 219.8 per 100,000 births), while the rate of congenital syphilis in newborn MHS beneficiaries increased by 355% (n=9 to n=32, 6.8 to 30.8 per 100,000 live births). Pregnant active component service members generally evinced higher rates of maternal syphilis than pregnant non-service member MHS beneficiaries during the reporting period. Additionally, the positive predictive value of maternal syphilis cases in the MHS was found to be low (59%). Future studies could focus on potential misclassification of maternal syphilis cases as well as syphilis screening compliance and treatment during pregnancy for all pregnant MHS beneficiaries.

**What are the new findings?:**

Rates of maternal and congenital syphilis increased from 2012 through 2022 within the Military Health System population. The rate of diagnosed maternal syphilis among pregnant female active component service members exceeded previously reported rates of syphilis among all female active component service members annually between 2015 and 2022, likely due to increased screening.

**What is the impact on readiness and force health protection?:**

Maternal and congenital syphilis affect the quality of life of service members and their families, as syphilis can lead to miscarriage and stillbirth in pregnant women and serious health conditions, even death, in newborns. Additionally, maternal, and congenital syphilis can increase Military Health System costs for short- and long-term treatments, and active component service members may experience increased lost duty days while managing their own or their beneficiary’s condition.

## 1 BACKGROUND

Syphilis is a sexually transmitted infection (STI) that can be spread by mother to fetus, which is called congenital syphilis, that can cause premature birth, low birth weight, miscarriage, stillbirth, and death of the newborn after birth.^1,2,3^ Congenital syphilis can also cause health problems in newborns such as deformed bones, anemia, enlarged liver and spleen, jaundice, nervous system complications, and meningitis.^1^

Congenital syphilis can be prevented through maternal syphilis detection from screening women during pregnancy.^1,2^ In 1996, the U.S. Preventive Services Task Force (USPSTF) began recommending routine screening for syphilis infection in all pregnant women.^4^ Since then, screening for syphilis during an initial pregnancy visit has been a well-established standard of care endorsed by the American College of Obstetricians and Gynecologists (ACOG), American Academy of Pediatrics (AAP), and American Academy of Family Physicians (AAFP).^5,6,7,8^ Additionally, the Department of Veterans Affairs (VA)/Department of Defense (DOD) clinical practice guideline also includes syphilis screening as a prenatal laboratory test recommended for routine pregnancy care.^9^ As of November 2023, syphilis screening during the first pregnancy visit was required by law in every state except Wisconsin, North Dakota, New Hampshire, Minnesota, Maine, Iowa, and Hawaii.^10^

In April 2024, the ACOG updated its recommendation to include serological screening of all pregnant individuals for syphilis at the first prenatal care visit, followed by universal re-screening during the third trimester and again at birth.^6^ Previous ACOG guidance had recommended risk-based testing in the third trimester only, for individuals living in communities with high rates of syphilis and those at risk of syphilis acquisition during pregnancy.^5^

Despite robust guidance for syphilis screening, in November 2023 the Centers for Disease Control and Prevention (CDC) published a Morbidity and Mortality Weekly Report (MMWR) about the increase in congenital syphilis cases in the U.S. between 2012 and 2022.^11^ Using its Notifiable Diseases Surveillance System (NNDSS), the CDC identified 3,761 cases of congenial syphilis in 2022—and determined that the number of congenital syphilis cases in the U.S. increased by 755% between 2012 and 2021.^11^ Along with this increase in congenital syphilis cases during this period, the CDC also found increased rates of primary and secondary syphilis in women 15-44 years of age between 2012 (2.1 per 100,000 population) and 2022 (19.1 cases per 100,000 population).^11^

In recent years, the rate of syphilis has increased among female active component service members (ACSMs). The June 2024 issue of *MSMR* reported on the incidence rate of syphilis between 2015 and 2023.^12^ The Armed Forces Health Surveillance Division (AFHSD) found the rate of syphilis in female ACSMs increased from 30.0 per 100,000 in 2015 to 80.4 per 100,000 in 2023, an increase of over 160% in 9 years.^12^ That report did not, however, address cases of maternal or congenital syphilis, and due to that knowledge gap, this report aimed to find the rate of maternal syphilis and congenital syphilis in Military Health System (MHS) beneficiaries receiving direct care through military hospitals and clinics or the private sector care between 2012 and 2022.

## 2 METHODS

Data for this study were derived from the Defense Medical Surveillance System (DMSS), a longitudinal database that includes records of both direct and privately sourced ambulatory health care encounters and hospitalizations of MHS beneficiaries in military hospitals and clinics as well as civilian (if reimbursed through MHS) treatment facilities worldwide. DMSS also includes records of reportable medical events (RMEs) from the Disease Reporting System internet (DRSi). The surveillance period was from January 1, 2012 through December 31, 2022.

The analysis was segmented in 2 categories: maternal syphilis and congenital syphilis. Maternal cases of syphilis were identified among MHS beneficiaries who had a live or stillbirth delivery from 2012 through 2022. Deliveries were identified by an inpatient or outpatient encounter for infant delivery recorded with an International Classification of Diseases, 9th Revision, Clinical Modification (ICD-9-CM) or 10th Revision, Clinical Modification (ICD-10-CM) code in any diagnostic position of the medical encounter (**Table**). A 280-day incidence rule was applied to identify separate delivery events; this methodology has been used as an estimate of gestational periods in previous analyses both in and outside the MHS.^14,15,16,17^ Age, beneficiary type, and race or ethnicity were identified at time of delivery. Maternal cases of syphilis were defined by a confirmed RME for syphilis or an inpatient or outpatient encounter with a syphilis diagnosis in any diagnostic position (**Table**). Diagnoses for early syphilis, late syphilis, and other and unspecified syphilis were included, as well as diagnoses for syphilis complicating pregnancy and childbirth. The syphilis diagnosis or RME was required to occur within 280 days on or prior to the delivery event to capture diagnoses during a pregnancy. Rates of maternal syphilis were calculated per 100,000 births.

The second part of this analysis evaluated rates of congenital syphilis. Cases of congenital syphilis were identified by a confirmed RME record for congenital syphilis or an inpatient or outpatient encounter with a congenital syphilis diagnosis in any diagnostic position (**Table**). These diagnoses were listed in the newborn’s medical record, and not linked to delivery events. The encounter had to occur in a neonate less than 28 days old, however. Live births from 2012 through 2022 were used as the denominator in rate calculations. Deliveries were excluded if a delivery encounter contained a diagnosis specific to stillbirth (ICD-9-CM: V27.1, V27.4, V27.7; ICD-10-CM: Z37.1, Z37.4, Z37.7). Rates of congenital syphilis were calculated per 100,000 live births.

Due to concerns that maternal and congenital syphilis cases could be overestimated by the methodology employed for this report, because of possible medical encounter coding of previous syphilis cases as if they were new cases, a random sample of 25 (2%) maternal syphilis cases and 20 (10%) congenital syphilis cases were retrieved for medical chart reviews. The sample sizes were chosen in accordance with the feasibility of the authors’ completion of the selected number of chart reviews. Cases in the sample were required to have been identified by ICD-9-CM or ICD-10-CM codes, and the care for the case had to be in an MHS hospital or clinic. Of those cases, medical charts could only be retrieved for 17 maternal and 11 congenital cases. Authors with clinical backgrounds conducted the chart reviews to confirm maternal and congenital syphilis diagnoses, looking to confirm that laboratory testing was positive for syphilis, maternal and congenital cases, and that maternal cases did not have a history of syphilis prior to pregnancy. These cases were used to calculate the positive predictive value (PPV) of the maternal and congenital syphilis case definitions. The PPV is the proportion of syphilis cases identified by the case definition employed by this report that were validated as true positive syphilis cases through medical chart review.

## 3 RESULTS

**Rates of maternal syphilis in the MHS**

**Figure 1:**
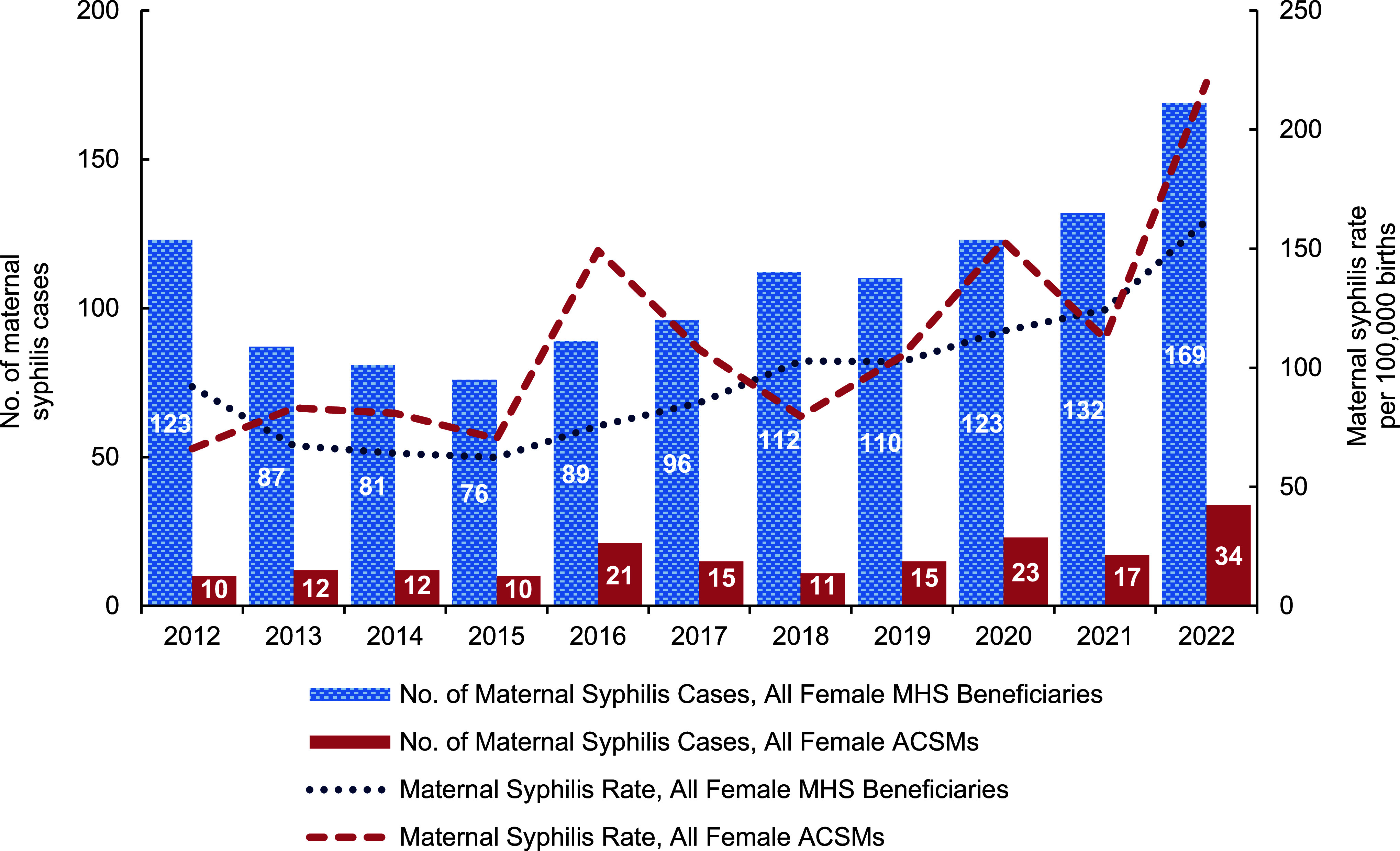
Cases and Rates of Maternal Syphilis During Pregnancy Among Female MHS Beneficiaries and Female ACSMs, 2012–2022

The rate of maternal syphilis during pregnancy generally increased between 2012 and 2022 among female MHS beneficiaries, with an overall rate of 94.0 cases per 100,000 births (n=1,198). While the number of births among female MHS beneficiaries decreased steadily from 2012 (n=133,590) to 2022 (n=104,475), the number of maternal syphilis cases increased (n=123 to n=169). Although the rate decreased from 2012 until 2015, it increased after 2015 and continued to rise annually through 2022 (**Figure 1**). Since 2015, the rate of maternal syphilis increased by 159%. In addition, the rate of maternal syphilis in female MHS beneficiaries increased by 30.1% between 2021 and 2022 alone. Between 2012 and 2022, maternal syphilis rates were highest among beneficiaries under 20 years of age (140.1 per 100,000 births) and among non-Hispanic Black or African Americans (182.5 per 100,000 births) (data not shown).

When the rate of maternal syphilis during pregnancy was calculated for female ACSMs only, the rate for ACSMs was higher than the rate for all female MHS beneficiaries during all but 3 years during the analysis period (2012, 2018, 2021) (**Figure 1**). Unlike the decrease seen in births among all female MHS beneficiaries between 2012 and 2022, births among female ACSMs remained stable during the study period (range 13,833–15,470 births) (data not shown).

**Rates of congenital syphilis in newborns in the MHS**

**Figure 2:**
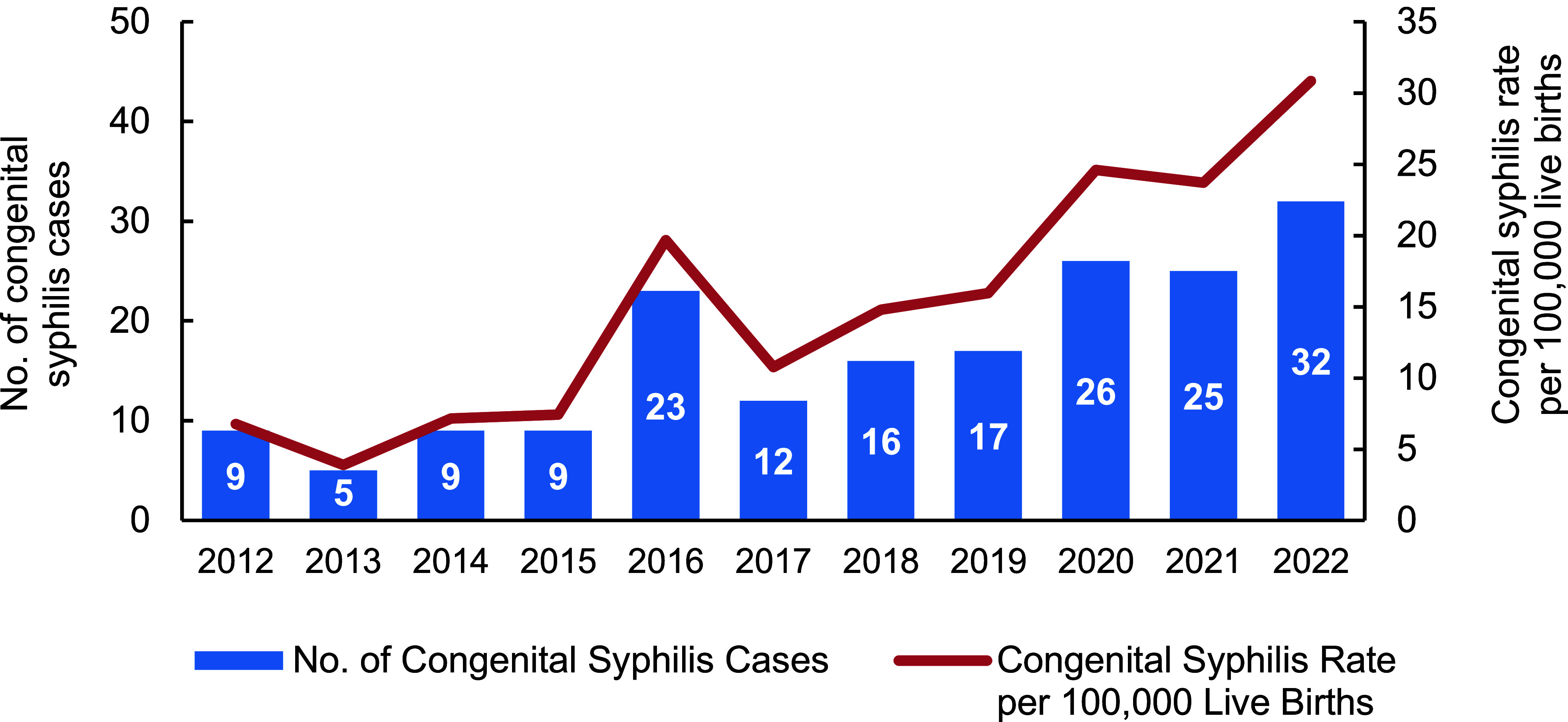
Cases and Rates of Congenital Syphilis Among Newborn MHS Beneficiaries, 2012–2022

The number of live births in the MHS population decreased between 2012 (n=132,900) and 2022 (n=103,753), while the number of congenital syphilis cases increased (n=9 to n=32). Those trends translate to an increasing rate of congenital syphilis in newborns in the MHS between 2012 and 2022, from 6.8 to 30.8 cases per 100,000 live births (**Figure 2**). Since 2017, the rate of congenital syphilis increased by 187%. Between 2021 and 2022 alone, the rate of congenital syphilis in newborn MHS beneficiaries increased by 30.1%; this is the same percentage increase seen in maternal syphilis among female MHS beneficiaries between 2021 and 2022.

**Positive predictive values of maternal and congenital syphilis**

The PPVs of syphilis differed in the maternal and congenital samples. Of the 17 maternal syphilis cases receiving a medical chart review, 10 cases were validated as true positive cases of incident syphilis diagnosed during pregnancy, per laboratory test results for syphilis and the additional criteria set forth in this study: a PPV of 59%. By contrast, of the 11 congenital syphilis cases receiving a medical chart review, 10 were validated as true positive cases of syphilis, per laboratory test results: a PPV of 91% (data not shown). A lower PPV suggests more false positive syphilis cases were found from the case definitions used to determine the maternal and congenital syphilis rates in this report.

## 4 DISCUSSION

Based on the data from the CDC and AFHSD, maternal and congenital syphilis is increasing in both the general U.S. and MHS populations. This new analysis adds to the Defense Health Agency’s knowledge on the rates of maternal and congenital syphilis, which were previously not reported separately from syphilis rates among female ACSMs.

While the CDC did not report on maternal syphilis rates specifically, they have reported increases from 2012 to 2021 of primary and secondary syphilis among U.S. women ages 15-44 years; rates of syphilis increased by 676% during that period.^11^ Within the MHS, maternal syphilis rates increased by 233% from 2012 through 2022 for pregnant, female MHS beneficiaries. Additionally, the rate of maternal syphilis among pregnant, female ACSMs exceeded the rate of syphilis among all female ACSMs annually between 2015 and 2022, as reported in the June 2024 *MSMR*.^12^ This difference was likely due to the additional syphilis screening pregnant women must undergo per ACOG, AAP, and AAFP guidance.

The CDC reported an increase of 31.7% in congenital syphilis cases in newborns in the U.S. between 2021 and 2022,^11^ while this analysis found a similar increase of 30.1% in rates of congenital syphilis in newborns within the MHS between 2021 and 2022. Additionally, the CDC reported an increase of 755% in congenital syphilis cases between 2012 and 2021,^11^ while this analysis found a smaller, yet significant, increase in congenital syphilis rates among newborns in the MHS at 355% between 2012 and 2022.

Due to concerns about potential misclassification of maternal and congenital syphilis cases using ICD-9-CM and ICD-10-CM codes, chart reviews were completed for a sample of 17 maternal and 11 congenital syphilis cases across the study period to assess case definition validity. The chart reviews of available medical records revealed that most cases of syphilis in newborns seen at military hospitals and clinics identified by the case definition (PPV 91%) were true cases of syphilis. Chart reviews also revealed that 82% (n=9) of the pregnant mothers of those congenital syphilis cases were screened, and 64% (n=7) were treated for syphilis during the pregnancy. This review suggests that the subsequent congenital syphilis diagnoses were due to either a treatment failure or were treated out of abundance of caution rather than a failure to screen and offer treatment to a pregnant mother. In cases when a pregnant mother was not treated before delivery, it was a result of no prenatal care, a loss to follow-up, or the mother declining treatment.

Conversely, only 59% of the syphilis cases identified in pregnant female MHS beneficiaries were true cases, which suggests that the maternal syphilis incidence data presented herein should be interpreted with caution, as they overestimate the true number of cases. This may be due, in part, to women’s incident diagnoses occurring prior to pregnancy, or false positive test results. The screening and confirmation of syphilis cases is complicated and can easily be misinterpreted through diagnostic codes alone. There is still an increasing trend over time, as the chart review did not suggest that case misclassification became better or worse over time.

Furthermore, the chart reviews found that pregnant women and newborns were generally appropriately diagnosed and treated for syphilis with antibiotics based on maternal and newborn history, physical examination, laboratory test results, and newborn radiography, when clinically indicated. Providers appear to have been using an abundance of caution in treating pregnant women and newborns for any possible syphilis infection when there was uncertainty, including using syphilis diagnostic codes when there was a history of syphilis prior to pregnancy. Providers’ continued adherence to DOD, ACOG, AAP, and AAFP guidance to properly screen, detect, and treat cases of syphilis supports the health and well-being of pregnant women and newborns. Additional chart reviews in future studies could more accurately calculate the potential overcounting of syphilis per the surveillance case definition.

The exact reasons for the increase in maternal and congenital syphilis from 2012 to 2022 among MHS beneficiaries cannot be determined by this analysis alone. Factors cited for the increase in the U.S. population include lack of adequate and timely testing and treatment, social and economic factors, and lack of awareness about the disease.^2,3,11^ Further analyses would be required to understand which, if any, of those factors are limitations shared in the MHS.

It is presumed that most, if not all, providers caring for pregnant patients in military hospitals and clinics are meeting the standard of care by screening for syphilis during the initial pregnancy visit. Increased compliance with syphilis screening guidelines may be a contributing factor to the increased numbers of maternal and congenital syphilis cases identified in this report. Additional analysis of syphilis screening compliance, as well as treatment administration and adherence, is warranted, however, to determine how these factors may have contributed to the increased rates of maternal and congenital syphilis. Additionally, a larger sample of case reviews could provide useful information on potential misclassification of maternal and congenital syphilis cases. Continued surveillance of syphilis cases in pregnant women and newborns and associated research on the impact of syphilis to military readiness is essential for understanding the true cost and burden to individuals in addition to the MHS, including increased medical resources and time military beneficiaries may have to interrupt their duties to attend appointments or care for affected family members.

## Figures and Tables

**Table 1:** ICD-9-CM and ICD-10-CM Diagnoses for Newborn Deliveries and Syphilis

Description	ICD-9-CM	ICD-10-CM
Newborn diagnoses		
Outcome of delivery	V27^a^	Z37^a^
Normal delivery	650^a^	O80
Cesarean delivery without mention of indication	669.7^a^	O82
Syphilis diagnoses		
Maternal syphilis	091^a^-097^a^, 647.00-647.03	A51^a,b^-A53^a^, O98.11^a^, O98.12
Congenital syphilis	090^a^	A50^a^

Abbreviations: ICD-9-CM, International Classification of Diseases, 9th Revision, Clinical Modification; ICD-10-CM, International Classification of Diseases, 10th Revision, Clinical Modification.

^a^Indicates that subsequent digits/characters are included.

^b^ICD-10-CM code A51.31 (condyloma latum) was excluded due to previously documented evidence of incorrect use of this code to diagnosis condyloma acuminata.^13^

## AUTHOR AFFILIATIONS

Defense Centers for Public Health–Aberdeen, Armed Forces Health Surveillance Division, Defense Health Agency, Aberdeen, MD: Ms. Kotas, LTC Yun, Dr. McCannon, Dr. Ambrose; Epidemiology and Analysis Branch, Armed Forces Health Surveillance Division, Silver Spring, MD: Dr. Stahlman; Defense Centers for Public Health–Falls Church, Armed Forces Health Surveillance Division, Falls Church, VA: Dr. Ying
